# The research trends of ferroptosis in diabetes: a bibliometric analysis

**DOI:** 10.3389/fpubh.2024.1365828

**Published:** 2024-03-06

**Authors:** Liyuan Xiong, Faquan Hu, Zhengpin Li, Xuemei Zhou, Yujiao Zheng

**Affiliations:** College of Traditional Chinese Medicine, Anhui University of Chinese Medicine, Hefei, China

**Keywords:** diabetes mellitus, ferroptosis, mechanism, bibliometric analysis, CiteSpace

## Abstract

**Objective:**

Exploring the mechanism of ferroptosis as a potential avenue for investigating the pathogenesis and therapeutic outlook of diabetes mellitus and its complications has emerged as a focal point within recent years. Herein, we employ a bibliometric approach to delineate the current landscape of ferroptosis research in the context of diabetes mellitus. Our objective is to furnish insights and scholarly references conducive to the advancement of comprehensive investigations and innovations in related domains.

**Methods:**

We included studies on ferroptosis in diabetes, obtained from the Web of Science Core Collection. All publications were transported in plaintext full-record format and were analyzed by CiteSpace 6.2.R4 for bibliometric analysis.

**Results:**

Four hundred and forty-eight records that met the criteria were included. The publications released during the initial 3 years were relatively small, while there was a sudden surge of publications published in 2022 and 2023. Representing 41 countries and 173 institutions, China and Wuhan University led the research on ferroptosis in diabetes. The author with the highest number of published papers is Zhongming Wu, while Dixon SJ is the most frequently cited author. The journal with the highest number of co-citations is *Cell*. The most common keywords include oxidative stress, cell death, lipid peroxidation, and metabolism. Extracted keywords predominantly focus on NLRP3 inflammatory, diabetic kidney disease, mitochondria, iron overload, and cardiomyopathy.

**Conclusion:**

The escalating recognition of ferroptosis as a potential therapeutic target for deciphering the intricate mechanisms underlying diabetes and its complications is underscored by a noteworthy surge in relevant research publications. This surge has catapulted ferroptosis into the spotlight as a burgeoning and vibrant research focus within the field.

## 1 Introduction

Diabetes has emerged as a rapidly escalating global health concern in the 21st century. According to data presented in the 10th edition of the Diabetes Atlas by the International Diabetes Federation, the global prevalence of diabetes is projected to reach 643 million by 2030 (1). By 2045, this figure is anticipated to surge to 783 million worldwide (2). Projections from the World Health Organization indicate that by 2030, diabetes will rank as the seventh leading cause of mortality globally (3). Hyperglycemia emerges as a cardinal symptom of diabetes mellitus, with prolonged elevation of blood glucose levels posing the risk of multi-organ damage, including the kidneys, eyes, and cardiovascular system (4). Consequently, the enduring presence of diabetes may precipitate a spectrum of grave complications, such as diabetic nephropathy (DN), diabetic neuropathy, and diabetic cardiovascular (DC) disease, significantly compromising patients’ quality of life. Given the escalating prevalence and mortality associated with diabetes, there exists an urgent imperative to discern efficacious therapeutic strategies targeting both diabetes and its attendant complications.

Ferroptosis, characterized by peroxidative damage induced by iron overload, manifests as cell membrane impairment leading to regulated cell death (5). In recent years, a plethora of studies have underscored the intricate association between ferroptosis and diabetes alongside its complications (6–15). Current research has elucidated the interplay between iron and glucose metabolism, highlighting their reciprocal influence. It is well-established that both iron deficiency and excess perturb glucose metabolism significantly (16), while conversely, heightened glucose levels precipitate iron overload, thereby instigating ferroptosis (17). Ferroptosis plays a pivotal role in the diagnosis, treatment, and prognosis of diabetes, emerging as a prominent topic in recent discussions on diabetes management. Recognized as a promising therapeutic target, a comprehensive understanding of ferroptosis’s pathogenesis and regulatory pathways is essential for the formulation of more efficacious treatment strategies. To delve deeper into the contribution of ferroptotic mechanisms to diabetes development, we conducted statistical and quantitative analyses utilizing bibliometrics. This methodology employs mathematical and statistical approaches to evaluate established and emerging research directions, encompassing co-authorship, co-citation, and co-occurrence analyses within a research area (18, 19). Through this bibliometric approach, we gained valuable insights into the current research landscape concerning ferroptosis in diabetes. This study aims to provide valuable ideas and references to facilitate in-depth research and development in related fields, offering essential guidance for future endeavors within this domain.

## 2 Materials and methods

### 2.1 Data source and search strategy

**Figure 1:**
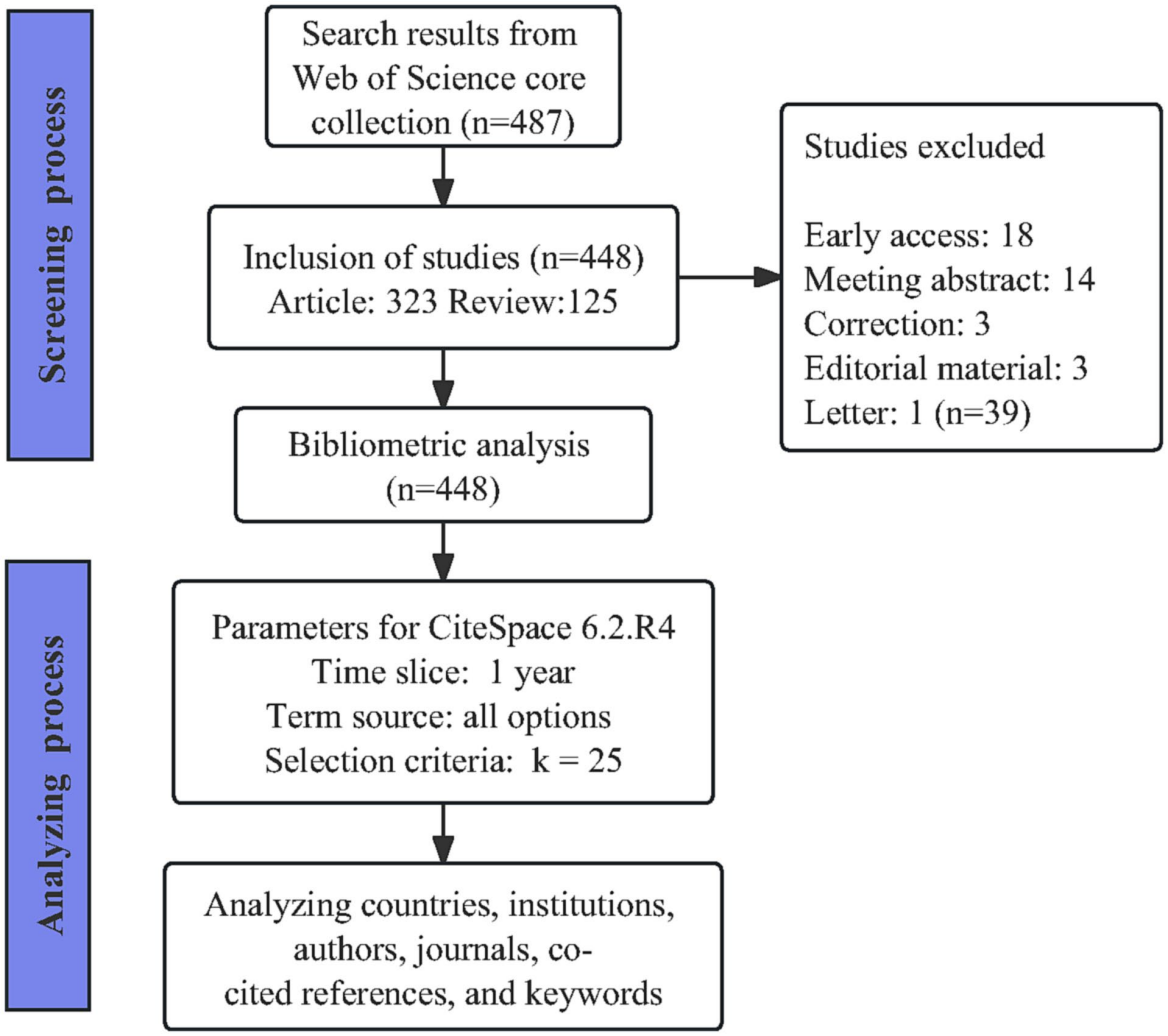
Research screening flow and bibliometric analysis methods.

The literature data was sourced from the Web of Science Core Collection (WOSCC), with the specific search retrieval formula detailed in the Supplementary material. Our study encompassed articles and reviews, with no restrictions on the start date of the search and a deadline set for November 2023. The language of the included literature was limited to English. The detailed screening and analysis process is depicted in Figure 1.

### 2.2 Data collection and analysis

Two authors systematically gathered literature from the WOSCC, adhering to predefined inclusion criteria, and exported it in a full-record plain text format. Any discrepancies were resolved through consensus during group consultation sessions. Subsequently, deduplication was performed using the built-in functions of CiteSpace 6.2.R4 software. Statistical analysis was conducted to assess the annual publication quantity and the distribution of publication types. Additionally, employing CiteSpace 6.2.R4 software, we conducted co-occurrence and cluster analyses on these documents across various dimensions, including country, institution, author, journal, co-cited reference, and keyword. This comprehensive analytical approach aims to provide a nuanced understanding of the current landscape within the field and to anticipate emerging research frontiers and hotspots in this area.

To delve into specific research trends, we utilized the capabilities of CiteSpace, a Java-based application tailored for visualizing and analyzing evolving patterns in published literature (20). This tool adeptly traces the trajectory of a particular research area over time, scrutinizing influential studies to pinpoint emerging trends and explore the frontiers of research (21). In the analysis of visualization plots generated by CiteSpace 6.2.R4, each node represents an analyzed factor, with its size proportional to item frequency. The thickness of lines connecting nodes indicates the strength of collaboration, while wide purple outer rings on nodes denote high centrality, indicating significant influence and robust collaboration. Nodes with centrality values exceeding 0.1 signify pivotal points in the field (22, 23). The clustering results obtained from CiteSpace provide Q-values and S-values, with notable clustering suggested when Q > 0.3, and substantial, reliable clustering indicated when S > 0.7.

## 3 Results

### 3.1 Trend analysis of publications

**Figure 2:**
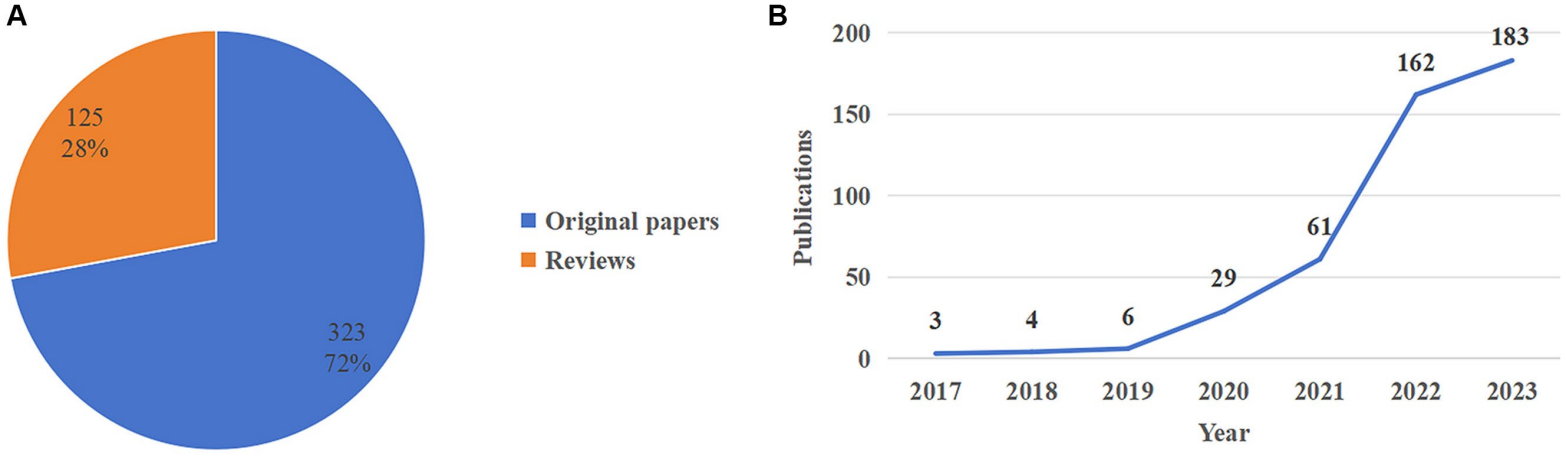
**(A)** The graph of the proportion of types of publications included shows a total of 323 original papers (72%) and 125 reviews (28%). **(B)** Trends in annual publications for studies of ferroptosis in diabetes. The horizontal coordinates of the graph represent time (year), and the vertical coordinates represent the number of publications.

We ultimately included a total of 448 research-related publications, comprising 323 original studies and 125 reviews (Figure 2A). According to the WOS citation report, literature concerning ferroptosis in diabetes made its debut in 2017. We meticulously documented the literature about ferroptosis in diabetes spanning from 2017 to 2023 (Figure 2B). Notably, there was a modest increase in annual publications from 2017 to 2019, totaling 13 publications. However, a significant surge occurred from 2020 onward, with 29 publications in 2020, a remarkable spike in annual publications in 2022, reaching 162, and a continued upward trajectory in 2023, with 183 publications. Research on ferroptosis in diabetes has witnessed a pronounced escalation, evolving into a prominent and emerging area of investigation within the field over the past 3 years. This trend signifies a growing interest in exploring preventive and therapeutic strategies for diabetes and its associated complications through the mechanism of ferroptosis, garnering increased attention from a burgeoning cohort of scholars.

### 3.2 Countries/regions analysis

**Figure 3:**
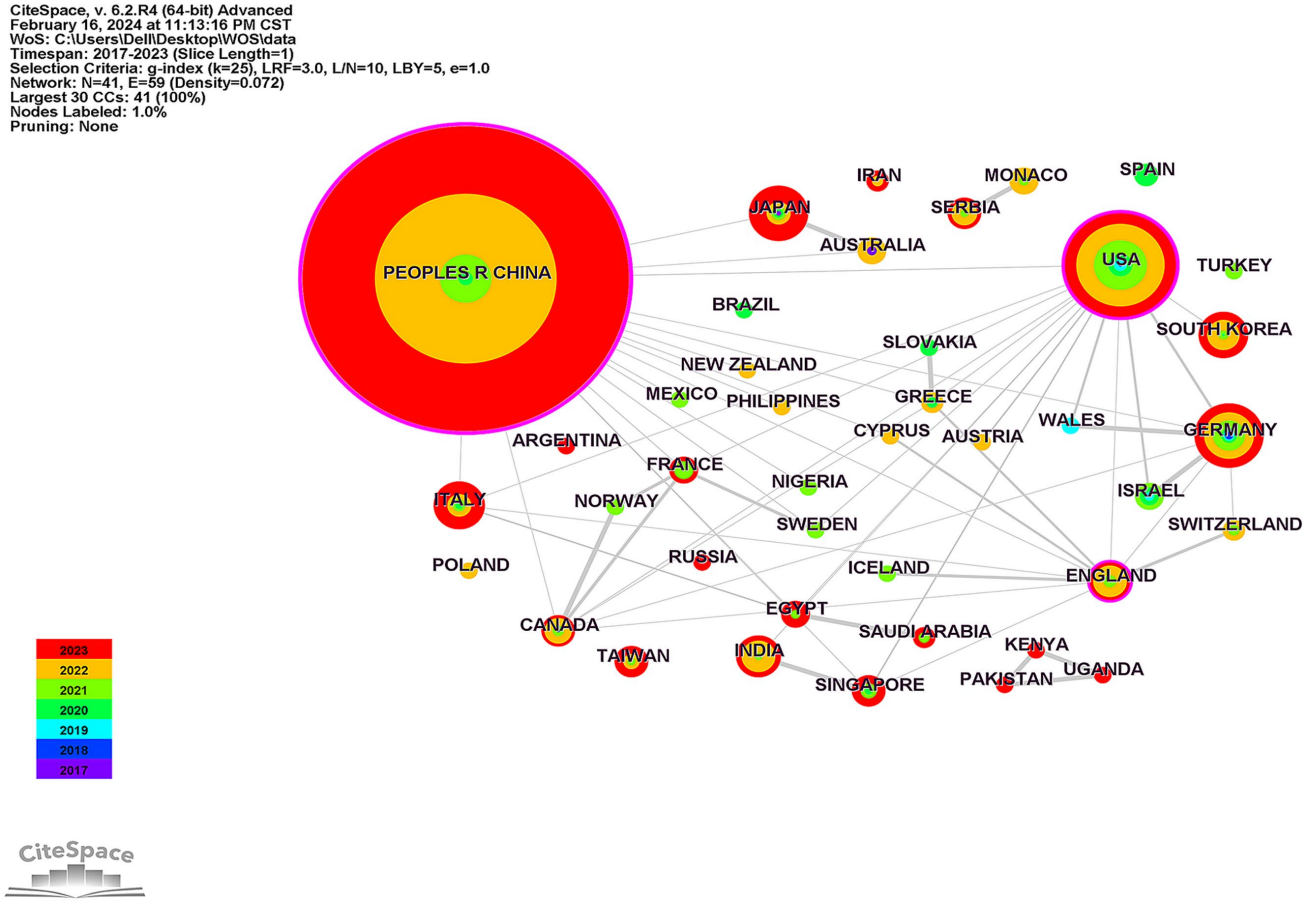
Visualization of national collaborative analyses related to the study of ferroptosis in diabetes. The nodes in the graph represent individual countries, the size of the nodes corresponds to the number of papers published in each country, and the thickness of the connecting lines indicates the strength of the collaborative relationship between the countries. The purple color around the node indicates high centrality, different colors of the nodes represent different publication years.

Between 2017 and 2023, research on ferroptosis in diabetes has garnered contributions from 41 countries and regions worldwide. These contributions have been visually mapped in Figure 3, which illustrates 41 nodes and 59 connecting lines. Table 1 presents the publications from the top 10 countries, with China leading the list with 353 publications, followed by the United States with 44 publications. Notably, these two countries significantly surpass others, underscoring their prominence in the field. Other countries within the top 10 include Germany, Japan, Italy, South Korea, India, and the UK, although none of them have contributed more than 20 publications individually. In the graphical representation, China exhibits the highest centrality at 0.44, closely followed by the US (0.31) and the UK (0.13). This suggests that China plays a pivotal role in international collaborations, highlighting its substantial research contribution to the global landscape of ferroptosis in diabetes. The size of nodes in the graph corresponds to the quantity of publications released by each country, while the thickness of connecting lines indicates the strength of collaborative relationships between countries. The visual depiction reveals extensive cooperation between China and the United States, as well as several other countries, including the United Kingdom, Germany, Canada, and France. In contrast, countries with fewer publications demonstrate less extensive collaboration.

**Table 1:** Top 10 countries and institutions with the highest number of publications.

Items	Rank	Name	Centrality	Year	Publications
Country/region	1	Peoples R China	0.44	2018	353
	2	USA	0.31	2017	44
	3	Germany	0.06	2017	18
	4	Japan	0	2017	12
	5	Italy	0	2020	10
	6	South Korea	0	2020	9
	7	India	0	2020	8
	8	England	0.13	2021	6
	9	Canada	0.05	2018	5
	10	Singapore	0.01	2020	4
Institution	1	Wuhan University	0.01	2020	18
	2	Central South University	0.14	2018	15
	3	Shanghai Jiao Tong University	0.09	2022	13
	4	Zhejiang University	0.04	2019	13
	5	Tianjin Medical University	0.03	2021	12
	6	Fudan University	0.15	2020	11
	7	Chinese Academy of Medical Sciences - Peking Union Medical College	0.21	2021	10
	8	Sun Yat Sen University	0.18	2022	10
	9	Huazhong University of Science & Technology	0.02	2020	10
	10	Nanjing Medical University	0.04	2022	9

### 3.3 Analysis of major institutions

**Figure 4:**
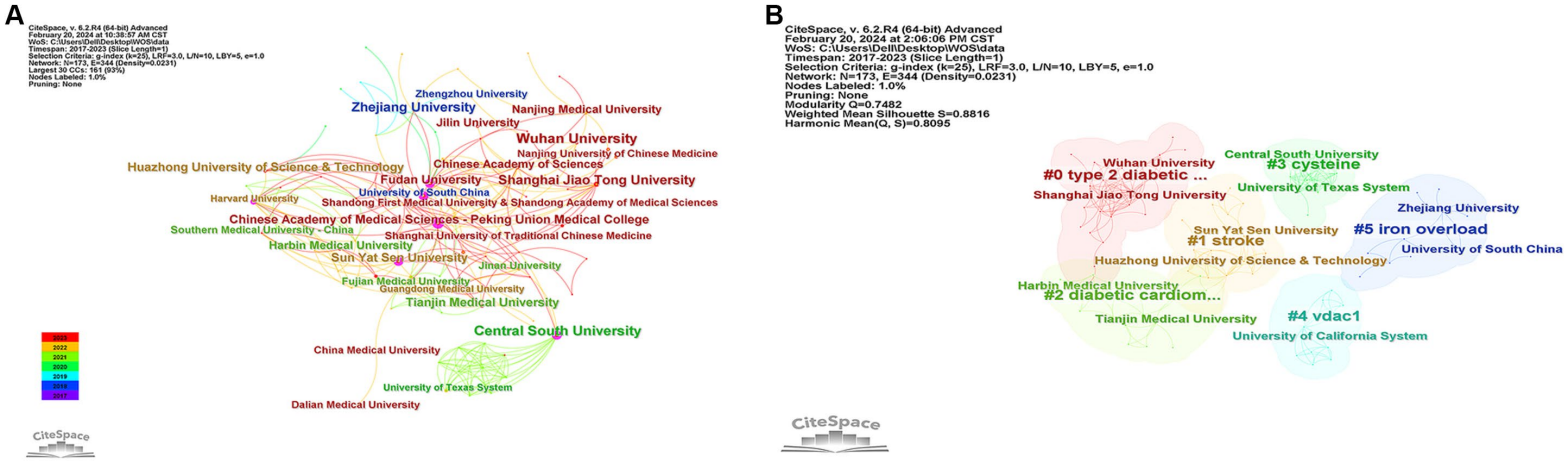
**(A)** Visualization of institution collaborative analyses related to the study of ferroptosis in diabetes. The institutions shown in the figure all have more than 5 publications, the nodes in the figure represent individual institutions, the size of the node corresponds to the number of papers published by each institution, and the thickness of the connecting lines at several points indicates the strength of the collaborative relationship between institutions. The purple color around the nodes indicates high centrality, different colors of the nodes represent different publication years. **(B)** Keyword cluster analysis for institutional research topics; Only the first six clusters are shown in the figure, and each cluster shows institutions whose publications are not less than 5, each node represents an institution, and different color groups represent different clusters, text with # indicates keyword clusters for these institutions, #0 = type 2 diabetic osteoporosis, #2 = diabetic cardiomyopathy.

Between 2017 and 2023, a total of 173 institutions have actively contributed to research on ferroptosis in diabetes. An extensive analysis of these institutions was conducted through visualization mapping (Figure 4A), revealing 173 nodes and 344 connecting lines. Table 1 provides details on the top 10 institutions by publication count, with Wuhan University leading with 18 publications, followed by Central South University with 15 publications, and Shanghai Jiao Tong University ranking third with 13 publications. Regarding institutional centrality, the Chinese Academy of Medical Sciences - Peking Union Medical College claimed the top spot with a centrality of 0.21, followed by Sun Yat Sen University (0.18), Fudan University (0.15), and Central South University (0.14). In the co-occurrence analysis of institutions, the dimensions of nodes in the graph correspond to the quantity of publications originating from each institution, while the thickness of connecting lines reflects the extent of collaboration between institutions. The study’s findings reveal that several institutions engage in extensive collaboration with others, including but not limited to the Chinese Academy of Medical Sciences - Peking Union Medical College, Wuhan University, Shanghai Jiao Tong University, Fudan University, Central South University, and Sun Yat-Sen University, among others. To investigate research themes across institutions, clustering analysis was conducted on the keywords found in publications from each institution, yielding highly credible results with *Q* = 0.7482 and *S* = 0.8816 in the top 6 clusters delineated by different color areas (Figure 4B). The largest cluster identified was #0 “type 2 diabetic osteoporosis,” followed by #1 “stroke.” Notably, the top three institutions are clustered in two subject areas, #0 “type 2 diabetic osteoporosis,” and #3 “cysteine,” highlighting their distinctive contributions to research in these domains.

### 3.4 Authors and co-cited authors analysis

**Figure 5:**
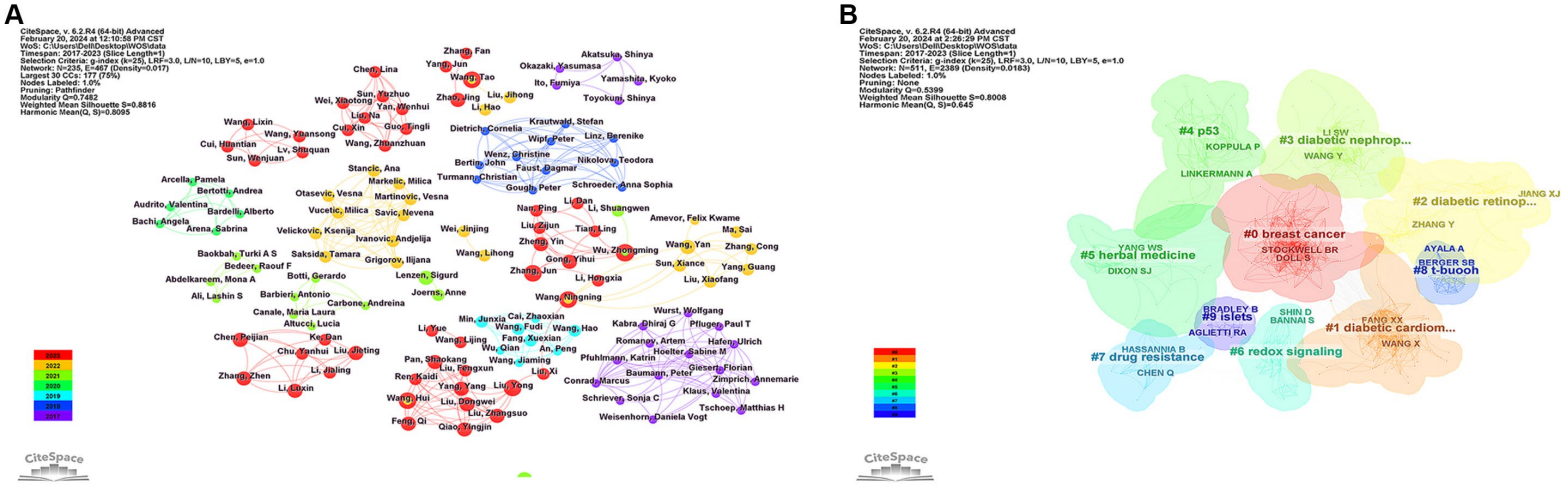
**(A)** Visualization of author collaborative analyses related to the study of ferroptosis in diabetes. Each node represents an author. The size of the node matches the author’s publication, the outer ring of the node appears in purple to indicate that the author has a greater centrality, and a separate region consisting of nodes and links indicates a collaborative relationship between authors, different colors of the nodes represent different publication years. **(B)** Keyword cluster analysis for co-cited authors’ research topics; only the first 10 clusters are shown in the figure, with only a portion of the influential authors shown in each cluster. Each node represents an author, and different color groups represent different clusters, text with # indicates keyword clusters for these co-cited authors, #1 = diabetic cardiomyopathy, #2 = diabetic retinopathy, #3 = diabetic nephropathy.

Between 2017 and 2023, a total of 235 researchers actively contributed to the study of ferroptosis in diabetes. We elucidated the researchers’ publication volume and collaborations through co-occurrence analysis (Figure 5A), which encompasses 235 nodes and 467 ties. Table 2 provides details on the top 10 authors by publication volume, with Wu Zhongming leading the list, closely followed by Liu Yong and Wang Hui, among others. It is noteworthy that the number of publications for each author is relatively small, implying considerable untapped research potential in the realm of diabetic ferroptosis studies. None of the top 10 authors exhibit high centrality in the plot, indicating decentralized group collaborations among them rather than centralized multi-crossover collaborations. In the analysis of citation frequency, co-citation relationships arise when two or more publications are simultaneously cited by other researchers. Table 2 delineates the top 10 researchers based on the frequency of co-cited authors. Further exploration of the literature authored by the most frequently cited researchers facilitates an understanding of research hotspots in the field. By clustering the keywords of publications authored by the co-cited authors (Figures 5B, *Q* = 0.5399, *S* = 0.8008), involving 511 co-cited authors. Among the first 10 keyword clusters, our analysis revealed that the top four authors, based on co-cited authors’ centrality, were Gao MH (0.07), Dixon SJ (0.06), Doll S (0.06), and Angeli JPF (0.06). Dixon SJ holds the highest citation count among the authors, with 247 citations. Cluster analysis indicates that this author’s primary research field is breast cancer, followed by Yang WS, whose research focus is herbal medicine. This analysis not only aids in grasping research hotspots but also provides guidance for researchers to conduct future targeted research in their respective fields.

**Table 2:** Top 10 authors and co-authors with the highest number of papers.

Items	Rank	Name	Centrality	Year	Publications/count
Author	1	Wu, Zhongming	0	2021	5
	2	Liu, Yong	0.01	2023	4
	3	Wang, Ningning	0.01	2022	4
	4	Zhang, Jun	0	2023	4
	5	Wang, Hui	0	2022	4
	6	Wang, Tao	0	2022	4
	7	Qiao, Yingjin	0	2023	3
	8	Zhao, Jing	0	2023	3
	9	Gong, Yihui	0	2023	3
	10	Pan, Shaokang	0	2023	3
Co-author	1	Dixon SJ	0.06	2017	247
	2	Yang WS	0.04	2018	162
	3	Stockwell BR	0.01	2019	148
	4	Doll S	0.06	2019	101
	5	Angeli JPF	0.06	2017	98
	6	Wang Y	0.01	2021	90
	7	Chen X	0.01	2020	89
	8	Gao MH	0.07	2018	84
	9	Li J	0.02	2020	81
	10	Fang XX	0.03	2020	78

### 3.5 Journals and co-cited journals analysis

Within the 448 publications exploring ferroptosis in diabetes, the most frequently cited papers appear in esteemed journals such as *Protein & Cell*, *Cellular & Molecular Immunology*, and *Free Radical Biology and Medicine*, among others. Notably, journals with the highest publication volumes include *Frontiers in Endocrinology* and *Oxidative Medicine and Cellular Longevity*, both of which play pivotal roles in advancing understanding of ferroptosis in the context of diabetes. Conducting a co-citation analysis of these journals helps elucidate their authority and influence in the field. Among the 532 cited journals, nine have amassed more than 200 citations. Table 3 delineates the top 10 co-cited journals with the highest citation counts. The most co-cited journal is *Cell*, boasting 329 citations, followed by *Free Radical Biology and Medicine* and *Cell Death and Disease*. The top three co-cited journals in terms of centrality are *Biochemical and Biophysical Research Communications* (0.07), *Free Radical Biology and Medicine* (0.05), and *Oxidative Medicine and Cellular Longevity* (0.05).

**Table 3:** Top 10 most co-cited journals involving ferroptosis in diabetes.

Rank	Co-cited Journal	Year	Count	Centrality	IF (2022)	JCR
1	Cell	2017	329	0.03	64.5	Q1
2	Free Radical Biology and Medicine	2017	296	0.05	7.4	Q1
3	Cell Death Disease	2018	257	0.04	9	Q1
4	Proceedings of the National Academy of Sciences of the United States of America	2017	232	0.02	11.1	Q1
5	International Journal of Molecular Sciences	2020	227	0.03	5.6	Q1/Q2
6	Nature	2017	221	0.01	64.8	Q1
7	Oxidative Medicine and Cellular longevity	2020	218	0.05	7.31 (2021)	Q2
8	Redox Biology	2017	215	0.03	11.4	Q1
9	Biochemical and Biophysical Research Communications	2017	203	0.07	3.1	Q3
10	Plos One	2017	197	0.01	3.7	Q3

**Figure 6:**
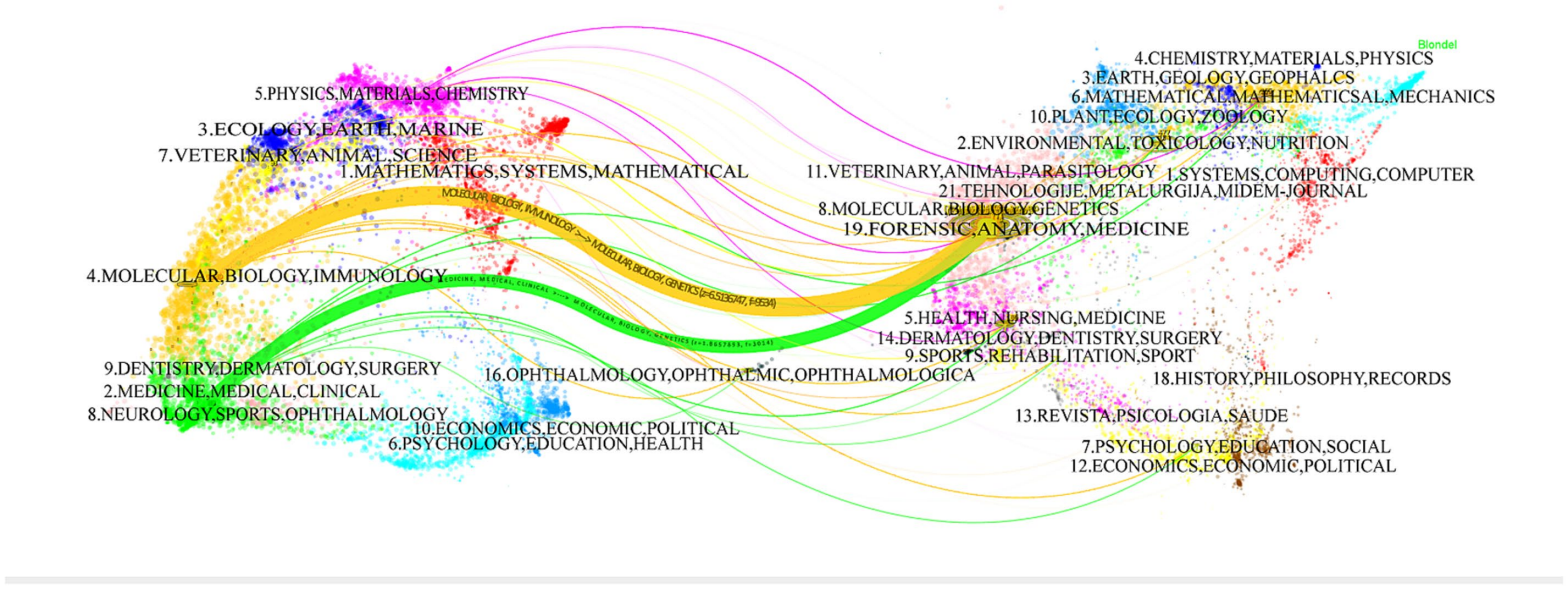
The dual-map overlay illustrates journals related to ferroptosis in diabetes, with citing journals on the left and cited journals on the right. The labels indicate the discipline, while the links represent the paths of citation.

The biplot overlay of journals illustrates the relationship between citing journals on the left side and cited journals on the right side, as depicted in Figure 6. Two main paths are discernible: the orange paths indicate that journals related to Molecular Biology, Genetics, Forensic Anatomy, and Medicine were cited by Molecular Biology and Immunology journals. The green paths suggest that journals related to Molecular Biology, Genetics, Forensic Anatomy, and Medicine were cited by Dentistry, Dermatology, Surgery, Clinical Medicine, Neurology, Sports, and Ophthalmology journals. This mapping reveals the cross-citation relationship between journals spanning various disciplines, reflecting the cross-collaboration and dissemination of knowledge within the research field.

### 3.6 Analysis of co-cited references and reference burst

**Figure 7:**
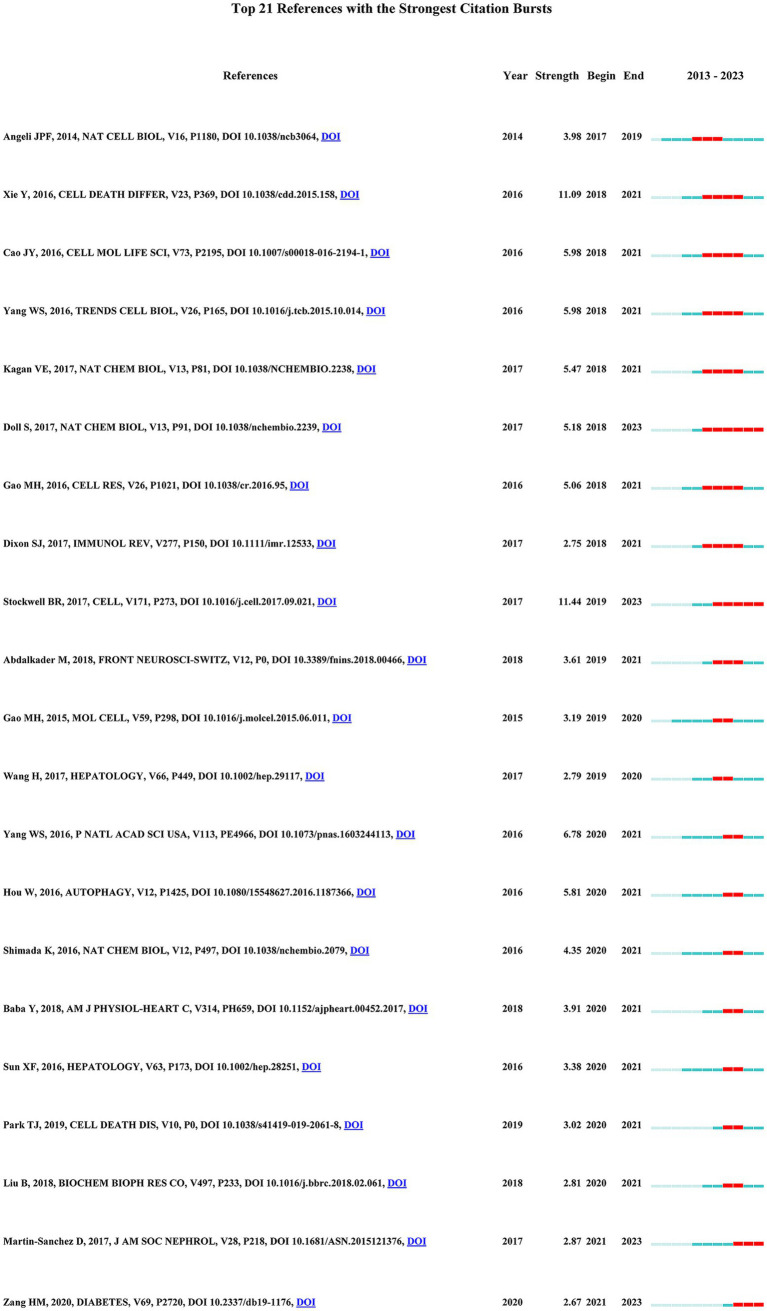
The top 21 references with the strongest citation bursts related to ferroptosis in diabetes.

From the 421 cited pieces of literature, we meticulously analyzed the citations that ranked in the top 10 based on co-citation frequency, as delineated in Table 4. Remarkably, the research conducted by Stockwell et al. (24) emerged as the most cited, garnering 76 citations, with an additional seven papers receiving citations exceeding 50 times. The majority of the top 10 co-cited research primarily delved into exploring the mechanisms underlying the initiation of ferroptosis, with particular emphasis on two pivotal regulatory factors, namely nuclear factor erythroid 2-related factor 2 (NRF2) and Ferroportin1 (FPN1). This observation underscores the paramount importance of these topics within the realm of diabetic ferroptosis research. Reference burst analysis proves invaluable in swiftly discerning the most influential cited literature from a vast array of references, thereby facilitating a clear understanding of the historical research landscape, as well as current cutting-edge research and emerging trends. In the reference burst analysis (Figure 7), higher strength values denote an explosive surge in the number of citations to the cited literature over a specific period. The work authored by Stockwell et al. (24) exhibited the highest burst intensity. This study primarily focuses on elucidating the mechanisms underlying ferroptosis, highlighting its relevance to the fields of biology and medicine, and providing essential tools and guidelines for studying this area. Consequently, it has established itself as a seminal work in the current research hotspots within the field. Doll et al. (25) and Stockwell et al. (24) represent two instances of literature outbreaks characterized by prolonged duration, indicating sustained activity and enduring influence within the research domain. Additionally, Stockwell et al. (24), Doll et al. (25), Martin-Sanchez et al. (26), and Zang et al. (27) stand out as recent studies within the last 3 years. Their primary focus lies in exploring the prevention of diseases related to ferroptosis through the inhibition of acyl-CoA synthetase long-chain family member 4 (ACSL4). Furthermore, these studies investigate the involvement of ferroptosis in model mice undergoing acute kidney injury, as well as examining the effects of ferroptosis mediated by NRF2 on myocardial injury in type 1 diabetes (25–27).

**Table 4:** Top 10 most co-cited references involving ferroptosis in diabetes.

Rank	Author	References	Citations	Centrality
1	Stockwell BR	Ferroptosis: A Regulated Cell Death Nexus Linking Metabolism, Redox Biology, and Disease	76	0.02
2	Li J	Ferroptosis: past, present and future	59	0
3	Li SW	Inhibition of ferroptosis by up-regulating Nrf2 delayed the progression of diabetic nephropathy	56	0.02
4	Fang XX	Ferroptosis as a target for protection against cardiomyopathy	55	0.05
5	Tang DL	Ferroptosis: molecular mechanisms and health implications	52	0
6	Bersuker K	The CoQ oxidoreductase FSP1 acts parallel to GPX4 to inhibit ferroptosis	50	0.04
7	Jiang XJ	Ferroptosis: mechanisms, biology and role in disease	50	0
8	Wang Y	Ferroptosis involves in renal tubular cell death in diabetic nephropathy	44	0.01
9	Doll S	FSP1 is a glutathione-independent ferroptosis suppressor	44	0.06
10	Dodson M	NRF2 plays a critical role in mitigating lipid peroxidation and ferroptosis	44	0.03

### 3.7 Analysis of frontiers and hotspots

**Figure 8:**
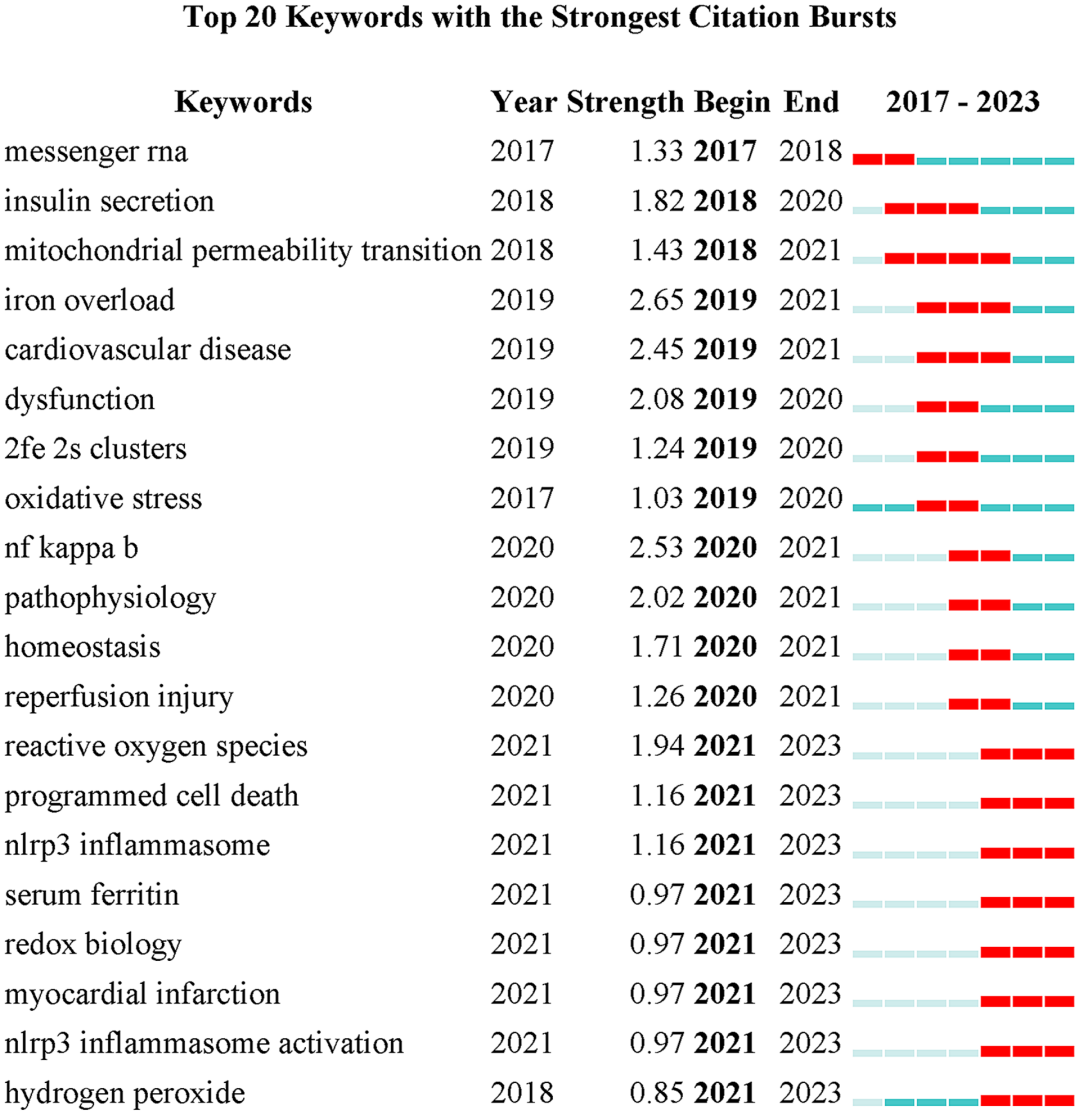
The top 20 keywords with the strongest bursts related to ferroptosis in diabetes.

**Figure 9:**
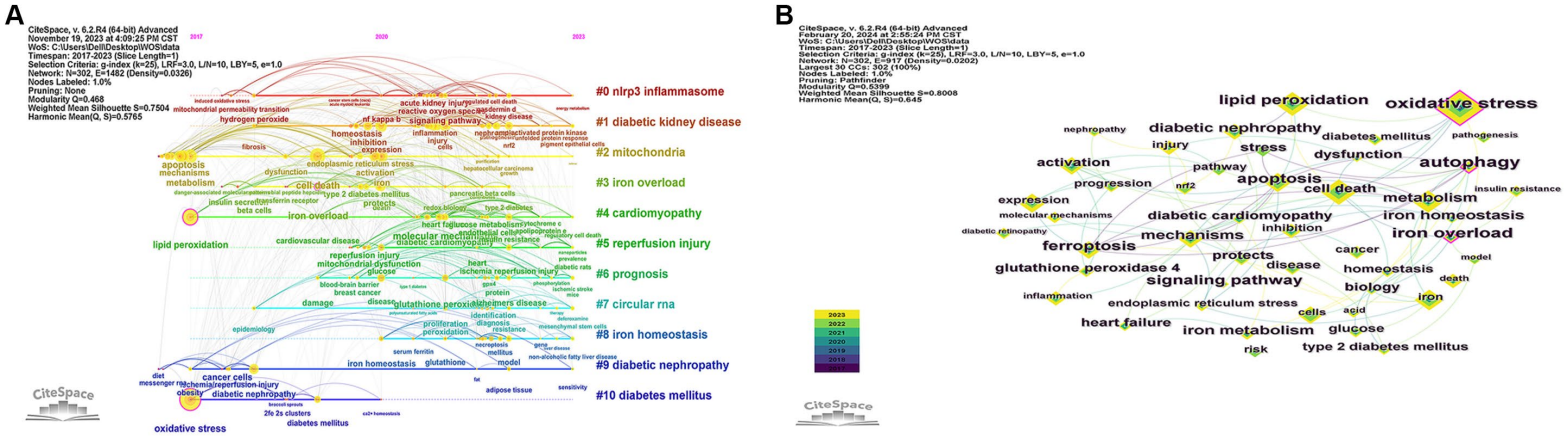
**(A)** Timeline view of keyword cluster analysis related to ferroptosis in diabetes. The top 11 clusters are shown in Figure 9A the names of the clusters are presented in sequential order on the right side, with keywords on the same horizontal line belonging to the corresponding cluster. Keywords are represented by nodes along the horizontal line, and the size of each node corresponds to the number of associated keywords, the position of the node on the horizontal line represents the year of the first appearance of the keyword. **(B)** Visualization of keyword co-occurrence related to ferroptosis in diabetes. The keywords shown in the graph all have a frequency of occurrence more than 10 times, each node represents a keyword, the size of the node is proportional to the size of the frequency of the keyword, and the thickness of the connecting line between the nodes represents the frequency of the two keywords appearing together, the multiple colors of the nodes represent the different years in which the keywords appear.

Keywords serve as crucial indicators of the focal points within a research domain. Figure 8 illustrates the top 20 keyword bursts analysis for ferroptosis in diabetes research. Burst strength is typically computed by amalgamating the frequency of keyword occurrences with the temporal aspect, where a higher strength value indicates a sudden or explosive surge in a keyword’s prevalence over a specified period. “Iron overload” emerges as the most prominent keyword, followed by “nuclear factor kappa b (NF-kappaB)” and “cardiovascular disease.” Notably, the keyword with the lengthiest burst duration was “mitochondrial permeability transition.” Keywords experiencing bursts in the last 3 years include “reactive oxygen species,” “programmed cell death,” “nod-like receptor family pyrin domain-containing 3 (NLRP3) inflammasome,” “serum ferritin,” “redox biology,” “myocardial infarction,” “NLRP3 inflammasome activation,” and “hydrogen peroxide.” This suggests that these topics currently constitute the forefront of research in the field. Following this, all keywords underwent clustering and analysis, displayed in a timeline view to observe the evolving research topics within the field of ferroptosis in diabetes over time. After conducting timeline analysis of keywords through CiteSpace, 14 clusters were identified, with Figure 9A illustrating the first 11 distinct clusters. In this analysis, there are 302 nodes and 1,482 connecting lines, with *Q* = 0.468 and *S* = 0.7504, indicating a significant clustering effect and credible results. The names of the clusters are presented in sequential order on the right side, with keywords on the same horizontal line belonging to the corresponding cluster. Keywords are represented by nodes along the horizontal line, with the size of each node corresponding to the number of associated keywords. The frequency of co-occurring keywords is indicated by the thickness of connecting lines between nodes. Timeline analysis indicates that the primary research focus to date has centered on the mechanism of diabetic ferroptosis, which plays a critical role in the progression of diabetes mellitus and its associated complications, garnering increasing attention from scholars. Figure 9B displays keyword co-occurrence, with the keyword “oxidative stress” holding the highest frequency at 136 times, ranking first, followed by “cell death,” “lipid peroxidation,” “metabolism,” “expression,” and “iron,” each occurring more than 50 times. Table 5 presents a compilation of the top 20 Keywords by frequency, embodying the focal points in the realm of ferroptosis research in diabetes.

**Table 5:** The 20 keywords with the highest frequency.

Rank	Keyword	Centrality	Year	Count
1	Oxidative stress	0.17	2017	136
2	Cell death	0.08	2019	85
3	Lipid peroxidation	0.11	2017	67
4	Metabolism	0.03	2017	60
5	Expression	0.03	2020	56
6	Iron	0.05	2020	51
7	Mechanisms	0.03	2017	49
8	Apoptosis	0.06	2017	47
9	Activation	0.03	2020	41
10	Ferroptosis	0.06	2017	41
11	Cells	0.01	2021	35
12	Inhibition	0.02	2020	33
13	Diabetic nephropathy	0.03	2018	32
14	Death	0.01	2020	31
15	Protects	0.03	2020	29
16	Injury	0.01	2021	27
17	Autophagy	0.08	2017	26
18	Cancer	0.01	2021	24
19	Inflammation	0.01	2021	24
20	Disease	0.03	2020	23

**Figure 10:**
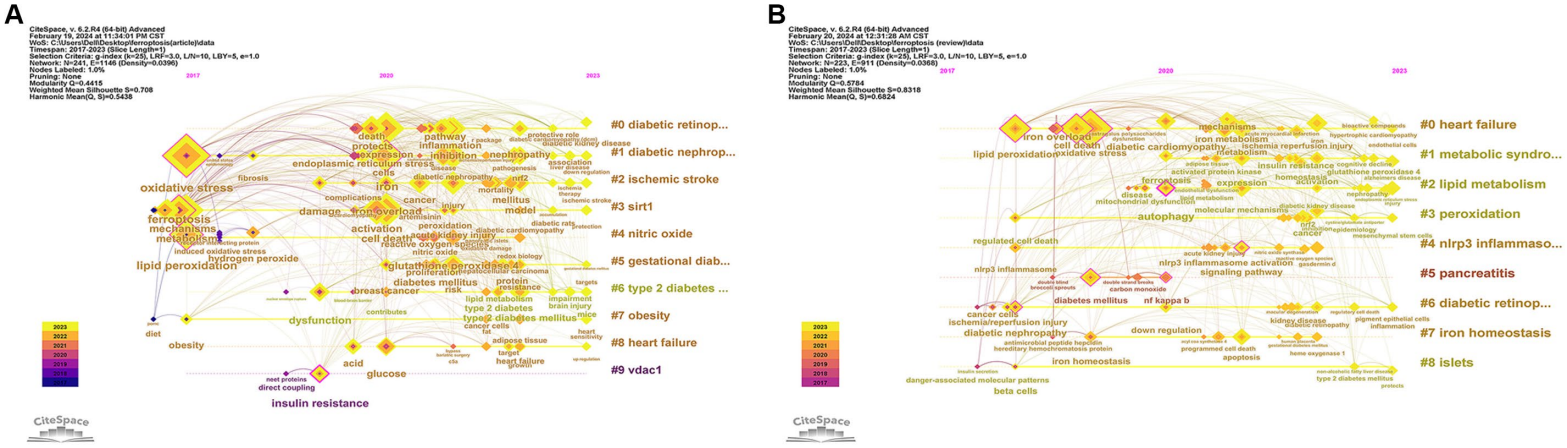
**(A)** Timeline view of the keyword cluster analysis of the original papers. The top 10 clusters are shown in Figure 10A #0 = diabetic retinopathy, #1 = diabetic nephropathy, #5 = gestational diabetes mellitus, #6 = type 2 diabetes mellitus. **(B)** Timeline view of the keyword cluster analysis of the reviews. The top 9 clusters are shown in Figure 10B #1 = metabolic syndrome, #4 = nlrp3 inflammasome, #6 = diabetic retinopathy. The names of the clusters are presented in sequential order on the right side, with keywords on the same horizontal line belonging to the corresponding cluster. Keywords are represented by nodes along the horizontal line, and the size of each node corresponds to the number of associated keywords, the position of the node on the horizontal line represents the year of the first appearance of the keyword.

Incorporating 323 original research articles and 125 reviews, we conducted a stratified analysis based on different publication types, uncovering distinct clustering keywords. Among the 323 original research articles, the timeline analysis of keywords is depicted in Figure 10A, showcasing the top 10 clusters. It was observed that original research predominantly focused on diabetic retinopathy (DR), DN, ischemic stroke, gestational diabetes, type 2 diabetes (T2D), obesity, heart failure, and other diseases. Additionally, two proteins, silent information regulator 1 (Sirt1) and Voltage-dependent anion-selective channel protein 1 (VDAC1), implicated in regulating diabetes, were identified. For the 125 reviews, the timeline analysis of keywords is presented in Figure 10B, displaying the top 9 clusters. Reviews mainly discussed heart failure, metabolic syndrome, pancreatitis, and DR, and also addressed the involvement of the NLRP3 inflammasome protein in diabetes regulation.

## 4 Discussion

### 4.1 General information

Upon scrutinizing the underlying data of this study, it becomes apparent that research within the realm of ferroptosis in diabetes was initially constrained during the inaugural three-year period. However, commencing in 2020, there has been a discernible surge in the volume of publications affiliated with this domain. Significantly, the years 2022 and 2023 have witnessed a pronounced escalation in the quantity of disseminated works, indicative of a burgeoning interest and scholarly investment in research endeavors pertaining to ferroptosis in the context of diabetes.

Among the included publications, 323 were research articles while 125 were reviews. Notably, China emerges as the foremost global contributor to the study of diabetic ferritin deposition, boasting an impressive tally of 353 publications. Following closely behind is the United States, positioned as the second significant contributor with 44 publications in this specialized field. Upon scrutinizing the cooperation network, it becomes evident that China and the United States have engaged in extensive collaborations with numerous other countries. However, the depth of this collaboration appears somewhat lacking. Conversely, countries such as Germany and Israel, Wales; Canada and Norway; Japan and Australia exhibit a higher intensity of cooperation, albeit with a narrower scope. This cooperative dynamic, while advantageous in certain respects, may not fully optimize the progress of research. Consequently, as endeavors are undertaken to broaden the scope of international collaboration, it becomes imperative to concurrently strengthen the depth of cooperation. Strategic leveraging of each participant’s strengths and advantages is paramount, fostering an environment conducive to exceptional achievements in this domain and facilitating an increase in the volume of published literature. Wuhan University emerges as a prominent institution with an extensive publication record in this field. Numerous studies originating from Wuhan University elucidate the potential amelioration of symptoms associated with diabetic complications by modulating ferroptosis through diverse pathways (12, 28–34). Among these, the two most cited studies from this institution delve into the implication of ferroptosis in cardiac injury induced by sepsis and diabetic myocardial ischemia/reperfusion injury (I/RI). Both studies propose symptom improvement through the modulation of ferroptosis mechanisms (12, 35).

The co-citation analysis identifies Gao MH (0.07) as the author possessing the highest centrality, indicative of substantial influence within the field and close associations with other authors. Regarding individual author contributions, posting frequencies are generally modest, with minimal variability observed among authors. Network analysis further reveals that a significant portion of authors operate within tightly-knit, smaller groups, suggestive of limited international collaboration. The collaborative landscape appears somewhat restricted, with certain authors lacking collaborative ties. Such circumstances may not be conducive to the progressive advancement of the field. Consequently, we advocate for authors to broaden the scope of their collaborations, facilitating knowledge exchange and fostering increased scholarly output within this domain.

Journals such as *Frontiers in Endocrine*, *Oxidative Medicine, and Cellular Longevity* distinguish themselves with their prolific publication records, underscoring their significant contributions to the advancement of research on ferroptosis in diabetes. Papers published in these journals, particularly those pertaining to endocrinology, oxidative medicine, and cytology, emerge as seminal references for contemporary studies within the field. Among co-cited journals, *Cell* stands out as the most frequently cited, indicating its pivotal role in the dissemination of research on this topic. The most cited research within the examined journals explores the functional interplay between the cystine transporter solute carrier family 7 member 11 (SLC7A11) and ferroptosis in the context of tumorigenesis. This study proposes therapeutic strategies based on its findings. Notably, the research highlights an association between the overexpression of SLC7A11 and increased tumor growth, suggesting a potential link between its promotion of tumorigenesis and the inhibition of ferroptosis (36). Furthermore, the dual-layered graph depicting journals elucidates the interdisciplinary character of this field, encompassing domains such as molecular biology, genetics, and anatomy, among others. Additionally, in the realm of clinical applications, disciplines including surgery, dermatology, and neurology reference this field. This multifaceted engagement across diverse disciplines underscores the field’s relevance in various clinical contexts, underscoring the ongoing imperative for the exploration and application of its mechanisms.

### 4.2 Hot topics and frontiers

The analysis of keywords is indispensable for encapsulating the fundamental facets of a research domain. In the burst analysis of keywords, “iron overload” emerges as the term exhibiting the highest burst intensity, underscoring its status as a focal point of current investigation. Concurrently, “mitochondrial permeability transition” boasts the longest burst duration, indicating sustained relevance within the field. Furthermore, “oxidative stress” garners attention due to its highest co-occurrence frequency, reflective of its historical significance in earlier research endeavors. In the timeline depiction of keywords (Figure 9A), recent research trajectories have centered on various diseases such as DN, diabetes mellitus, inflammation, and cancer. These pivotal terms are poised to maintain their prominence, indicative of ongoing and prospective areas of interest. Notably, the results of cluster analysis align with specific keywords. In the keyword clustering, cluster #0 delineates the NLRP3 inflammatory vesicle, recognized as a key regulator of ferroptosis. This aspect is under intensive scrutiny and validation in the current study to ascertain its significance within the field. DN emerges as a central focus within this research domain, notably underscored in clusters #1 (diabetic kidney disease) and #9 (diabetic nephropathy) during cluster analysis. As a significant microvascular complication of diabetes, DN holds clinical importance as a primary contributor to renal failure (37). Urgent preventive and early treatment strategies are imperative, thus highlighting its pivotal status in current research endeavors. Additionally, clusters #4 (cardiomyopathy), #5 (reperfusion injury), and #10 (diabetes mellitus) also denote key diseases and complications extensively investigated within the field. The cluster analysis further encompasses #2 (mitochondria) and #3 (iron overload module), both elucidating intricate mechanisms of ferroptosis. Understanding iron overload and mitochondrial homeostasis is crucial in comprehending ferroptosis dynamics. Clusters #6 (prognosis) and #8 (iron homeostasis) signify the attainment of iron homeostasis and the pursuit of a favorable prognosis as ultimate objectives within this research domain. Cluster #7 (circular RNA) sheds light on the role of gene regulation in initiating ferroptosis. Recent observations in the timeline view highlight a notable upsurge in interest in gene-related topics, particularly in the context of ferroptosis. Current research endeavors focus on elucidating genes associated with ferroptosis, such as ACSL4, glutathione peroxidase 4 (GPX4), SLC7A11, NRF2, NADPH oxidase 1 (NOX1), among others (38). As investigations into the mechanisms underlying ferroptosis progress, it is anticipated that additional regulatory genes and factors will be unveiled in the foreseeable future.

Mechanisms underlying ferroptosis exhibit nuanced variations in functionality across diverse disease contexts, presenting an avenue for potential intervention to ameliorate the impact of diabetes and its associated complications. Type 2 diabetes mellitus (T2DM) dominates the global prevalence of diabetes mellitus, manifesting a spectrum of complications, notably implicating the cardiovascular and renal systems (39). Despite advancements, the pathogenesis of diabetes remains incompletely understood, necessitating further validation in studies about ferroptosis. According to current research, ferroptosis manifests through elevated intracellular iron concentrations alongside the accumulation of lipid peroxides, culminating in pancreatic β-cell damage. This damage precipitates impaired insulin secretion from pancreatic β-cells. Consequently, a comprehensive exploration of ferroptosis-associated mechanisms holds the potential to enhance the diagnosis, treatment, and prognosis of T2DM (40). This area represents a current focal point in research, offering promising avenues for investigation.

Several research investigations have emphasized the pivotal role of ferroptosis in the pathogenesis of complications associated with diabetes (41). It is elucidated that in diabetic complications, hyperglycemia induces alterations in coregulators, resulting in iron overload and diminished antioxidant capacity (7, 42). Subsequently, this cascade leads to the accumulation of lipid peroxides within cells, ultimately culminating in ferroptosis (41). Furthermore, the hyperglycemic milieu induces oxidative stress, leading to an excessive generation of reactive oxygen species (ROS) and triggering mitochondrial fragmentation mediated by the mitochondrial fission regulator dynamin-related protein 1 (Drp1) (43). The compromised mitochondrial fragmentation exacerbates oxidative stress, thereby promoting ferroptosis. Notably, studies have provided evidence suggesting that activators of AMP-activated protein kinase (AMPK) can protect pancreatic β-cell mitochondria by inhibiting Drp1 activity (44). Additionally, it has been revealed that mitochondrial metabolism plays a significant role in regulating ferroptosis and possesses an inherent defense mechanism against it (45). In our keyword clustering analysis (Figure 9A), incorporating the modules “#2 mitochondria,” “#3 iron overload,” and “#8 iron homeostasis,” the maintenance of iron homeostasis and the restoration of mitochondrial function emerge as potential inhibitory factors against ferroptosis onset. Recent research has elucidated the intricate mechanisms underlying ferroptosis, implicating oxidative stress, iron overload, mitochondrial dysfunction, and inflammatory responses as key factors contributing to its occurrence. Nonetheless, numerous complexities within these mechanisms warrant further comprehensive investigation. Ferroptosis assumes a pivotal role across a spectrum of diseases, as underscored by highlighted studies in our keyword cluster analysis. Validating the involvement of ferroptosis across various diseases via diverse targets and exploring the therapeutic efficacy of different drugs through varied pathways constitute critical research endeavors with profound clinical implications. We have distilled select findings from investigations in targeted therapies for diseases, accentuating the current research priorities and prospective frontiers in this burgeoning field.

#### 4.2.1 Molecular regulation of ferroptosis based on findings

In the cluster analysis of the keywords (Figures 9A, 10B), all showed clustering of NLRP3 inflammatory vesicles, a combination of cited literature analysis and co-occurrence analysis of keywords revealed a high frequency of NRF2. Consequently, recent investigations into the management of diabetes mellitus and its associated complications have gravitated toward the exploration of NLRP3 inflammasome and NRF2. The NLRP3 inflammasome, a multiprotein complex localized within the cytoplasm, demonstrates aberrant activation closely intertwined with the pathogenesis of various inflammatory conditions. Recent investigations have elucidated the pivotal role of the NLRP3 inflammasome in the inflammatory response associated with the pathogenesis of diabetes mellitus. Moreover, emerging evidence has unveiled a significant association between NLRP3 inflammasome activation and the onset of ferroptosis (46). Specifically, data suggest that NLRP3 inflammatory vesicle-mediated pyroptosis substantially contributes to ferroptosis induced by T2D. A recent study has demonstrated that the depletion of cluster of differentiation 74 (CD74) effectively prevents T2D-induced cardiac remodeling and contraction dysfunction by modulating ferroptosis through the NLRP3/pyroptosis pathway (47). Additionally, Quagliariello et al. (48) have shown that Empagliflozin mitigates ferroptosis, fibrosis, apoptosis, and inflammation in adriamycin-treated mice by engaging the NLRP3-related pathway, consequently leading to a notable enhancement in cardiac function. Nevertheless, further investigation is warranted to delineate the regulatory role of the NLRP3 inflammasome in ferroptosis, thus providing novel therapeutic targets and strategies for NLRP3 inflammasome-associated disorders (46). Li et al. (10) have demonstrated that the progression of diabetic complications can be decelerated through the inhibition of ferroptosis via upregulation of NRF2. Sirt1, a nicotinamide adenine nucleotide (NAD)-dependent protein deacetylase, has been shown to positively modulate NRF2 expression and activity, thus impeding cellular ferroptosis (49). At the transcriptional level, NRF2 governs critical genes involved in iron storage and transportation (50), assuming a central role in antioxidative stress by orchestrating the expression of a repertoire of signaling proteins and enzymes, thereby preserving cellular redox equilibrium and oxidative milieu (51). Hence, NRF2 emerges as another pivotal therapeutic target for ameliorating complications associated with diabetes.

#### 4.2.2 Function of ferroptosis in diabetes and complications

##### 4.2.2.1 Function of ferroptosis mechanisms in diabetic nephropathy

Ferroptosis assumes a crucial role in the progression of complications related to diabetes. From this perspective, numerous crucial targets, pathways, and drugs for treating of complications related to diabetes can be identified. Keyword clustering analysis has revealed that DN is encompassed within two prominent clusters, designated as #1 and #9, rendering it a focal point of investigation within the diabetic complications domain. Given the dire consequences of DN in its advanced stages, there is an urgent imperative to identify effective therapeutic targets and agents. N-acetylcysteine has been posited as a potential candidate for mitigating DN by modulating ferroptosis through activation of the Sirtuin-3-Superoxide dismutase 2/GPX4 (SIRT3-SOD2/GPX4) pathway. Notably, N-acetylcysteine exhibits efficacy in bolstering antioxidant defenses and dampening inflammation in renal injury, while also targeting the major ferroptosis regulator, GPX4. Concurrently, the regulatory role of SIRT3 impacts mitochondrial oxidation, thereby modulating intracellular metabolism. Enhanced SIRT3 activity facilitates the conversion of superoxide, thereby ameliorating mitochondrial oxidative stress and contributing to the attenuation of ferroptosis (52). Furthermore, two independent studies have demonstrated the efficacy of calycosin and aspirin in modulating ferroptosis, manifesting a protective effect with favorable outcomes in DN (53, 54). Another investigation has elucidated that Schisandrin A, a constituent of Schisandra, inhibits ferroptosis in DN by targeting the AdipoR1 protein. This research underscores the therapeutic potential of Schisandrin A in ameliorating hyperglycemia-induced ferroptosis and ROS-mediated apoptosis, thereby attenuating oxidative stress and inflammatory responses (55). Recent inquiries have uncovered that Vitexin inhibits GPX4-mediated ferroptosis through both *in vivo* and *ex vivo* experiments, offering alleviation for DN. Vitexin, a monomeric flavonoid derived from anti-inflammatory and anticancer botanical sources, exhibits substantial nephroprotective effects by mitigating kidney injury and ferroptosis (56). In summary, the mitigation of DN can be accomplished by targeting ferroptosis mechanisms, highlighting the imperative for further exploration and development of diverse pharmaceutical agents in this realm.

##### 4.2.2.2 Function of ferroptosis mechanisms in diabetic cardiomyopathy

Keyword clustering analysis has revealed that cardiomyopathy, categorized as #4, constitutes a prominent cluster, emerging as a focal point in the investigation of diabetic complications. Multiple studies have reported significant improvement in DC through modulation of ferroptosis mechanisms. One study suggests that sulforaphane, an activator of NRF2, can mitigate DC by inhibiting ferroptosis via AMPK-mediated NRF2 activation (57). Several investigations have corroborated that curcumin, 6-gingerol, and dexmedetomidine attenuate ferroptosis, thereby mitigating myocardial injury induced by DC, through activation of the NRF2, NRF2/Heme Oxygenase-1 (HO-1), and NRF2/GPX4 pathways, respectively (58–60). These findings underscore the pivotal role of NRF2 in the context of DC, highlighting various pharmaceutical agents’ capacity to ameliorate DC by suppressing ferroptosis through NRF2 upregulation. Moreover, five bioactive compounds, namely ginsenoside Rg1, gypenosides, quercetin, ursolic acid, and salidroside, have demonstrated efficacy in ameliorating DC by inhibiting NLRP3 inflammatory vesicles. These natural agents exert significant inhibitory effects on diabetic myocardial fibrosis and inflammation, primarily through the suppression of NF-κB or ROS-mediated signaling pathways (61). This presents a promising avenue for future exploration, with the potential to unveil additional compounds capable of exerting their therapeutic effects via similar mechanisms.

##### 4.2.2.3 Function of ferroptosis mechanisms in diabetic reperfusion injury

Another module identified in the keyword clustering analysis revolves around “#5 reperfusion injury” (RI), which can manifest in multiple organs throughout the body. In our investigations, we observed that myocardial ischemia reperfusion injury (IRI) resulting from diabetes mellitus is particularly prevalent. Persistent hyperglycemia has been implicated in inducing vascular damage, leading to vasoconstriction and precipitating myocardial ischemia. Subsequent restoration of blood flow exacerbates RI, thus assuming significance in the context of diabetic complications. Li et al. (12) discovered that inhibition of ferroptosis could attenuate RI stemming from diabetic myocardial ischemia, offering a promising therapeutic target for myocardial ischemic disease. In a study conducted in 2022, it was demonstrated that activation of the NRF2/FPN1 pathway effectively regulates iron homeostasis and ferroptosis, thereby mitigating myocardial IRI in diabetic rats (33). Additionally, Huang et al. (28) illustrated that Nobiletin possesses the ability to inhibit ferroptosis and ameliorate myocardial IRI in rats with T2DM. Nobiletin, derived from citrus peels, is a flavonoid renowned for its anti-inflammatory and antioxidant properties. Collectively, the majority of the aforementioned drugs primarily exert their effects through antioxidant and anti-inflammatory mechanisms.

##### 4.2.2.4 Function of ferroptosis mechanisms in diabetic retinopathy

In the keyword clustering analysis encompassing original studies and reviews, DR has emerged as a significant focus of current investigations. As a critical complication of diabetes, DR poses a substantial risk of visual impairment and blindness, exhibiting severity ranging from non-proliferative DR to proliferative DR (62, 63). Moos et al. (64) have demonstrated that PMX500FI, a novel synthetic derivative of α-lipoic acid, exhibits the ability to activate the NRF2 signaling pathway and mitigate oxidative stress. This compound holds promise in attenuating DR by augmenting iron uptake and suppressing the generation of lipid peroxides (65). Additionally, other investigations have revealed the efficacy of strategies such as elabela and ferrostatin-1 in alleviating ferroptosis in the retina. These strategies have been validated through numerous cellular and animal experiments, indicating their mode of action via activation of the Cystine/glutamate Antiporter system XC (XC-)-GPX4 axis (66, 67). The study conducted by Liraglutide, a widely recognized antihyperglycemic agent, primarily modulates oxidative stress and endoplasmic reticulum stress. Its mechanism involves the augmentation of NRF2 and Thioredoxin (Trx) expression, thereby mitigating the pivotal role of ferroptosis in the progression of DR (68). Given the significant involvement of ferroptosis in the pathological process of DR, the imperative for developing pharmacological interventions capable of rectifying retinal ferroptosis is underscored.

In conclusion, Iron metabolism is involved in several processes of glucose metabolism in the body, including insulin secretion, hepatic metabolism, and fat metabolism, and maintains glucose homeostasis in several organs and tissues (69), furthermore, metabolism emerges as a primary focus in related studies, as delineated by our keyword analysis. Importantly, ferroptosis ensues in the presence of abnormal iron levels, exacerbating the progression of diabetes and its associated complications. Therefore, the pursuit of further investigations aiming to ameliorate diabetes and its complications by targeting ferroptosis mechanisms has emerged as a prominent and burgeoning research topic in this field. Recent studies have not only identified herbal and Western therapeutic modalities but also witnessed a surge in combined Chinese and Western treatment approaches. This trend underscores the active contribution of every medical discipline toward a comprehensive exploration of ferroptosis-targeted therapies in diabetes. Collectively, these studies suggest that ferroptosis, recognized as a potential therapeutic avenue for diabetic complications, holds significant promise in shaping future strategies for addressing diabetes and its associated complications. Numerous key pathways implicated in ferroptosis induction have been elucidated, encompassing factors such as iron overload, inhibition of SLC7A11, GPX4, NRF2 protein activity, and activation of NLRP3 inflammatory vesicles. However, the existence of additional crucial pathways in ferroptosis regulation remains uncertain. While our analysis has predominantly centered on ferroptosis in the context of diabetes, its significance extends to a multitude of diseases. For instance, our keyword and co-citation analysis underscore the pivotal role of ferroptosis mechanisms in tumor development, wherein *in vivo* inhibition of ferroptosis may paradoxically facilitate tumor growth. Furthermore, our findings reveal the involvement of ferroptosis in conditions such as Alzheimer’s disease and ischemic stroke. The elucidation of ferroptosis-related mechanisms across diverse disease states remains a focal point of ongoing research and development. Moving forward, further exploration and validation of the utility of ferroptosis mechanisms are imperative. In the realm of ferroptosis regulation, the capacity to selectively target specific cells emerges as a critical consideration for expanding the applicability of ferroptosis modulation. To delineate superior therapeutic approaches and provide precise clinical guidelines, future research endeavors will be directed toward consolidating and corroborating these emerging avenues.

### 4.3 Strengths and limitations

We are the pioneering article to undertake a bibliometric analysis of studies about ferroptosis in diabetes and meticulous examination delves into research hotspots and trends within the field, summarizing key findings and identifying research frontiers. This study intends to furnish valuable insights that can guide future research directions in this domain. However, several limitations should be acknowledged. Firstly, Ferroptosis represents an emerging area of study. In recent years, Though research on ferroptosis has increasingly focused on elucidating its biological mechanisms in various diseases, the number of studies specifically investigating ferroptosis in the context of diabetes remains relatively small. Moreover, our study relied exclusively on data obtained from the WOSCC, thereby potentially limiting the number of publications included and consequently influencing the robustness of our conclusions. Furthermore, the outcomes of our search are inevitably influenced by the selection of search terms, introducing a level of subjectivity to the results. The data analysis conducted in this article relied exclusively on CiteSpace software, which possesses inherent limitations in its application. Secondly, the quality of the literature included in our analysis was not systematically evaluated, potentially leading to biased outcomes. Moreover, delays in the publication of certain cited works may introduce inaccuracies in our assessment of high citation rates. It is imperative to acknowledge the limited temporal scope of our investigation, encompassing solely the years 2017 to 2023. To procure a more exhaustive and dependable comprehension, forthcoming inquiries ought to contemplate protracted, extensive-scale, multicenter studies to authenticate and amplify our discoveries.

## 5 Conclusion

We collected 448 publications from the WOSCC and utilized CiteSpace 6.2.R4 for visualization and analysis. Among these studies, contributions were observed from 44 countries, 173 institutions, and 235 authors in the realm of ferroptosis in diabetes. Notably, there has been a significant increase in research publications in this field over the past 3 years, indicating a burgeoning interest and activity in this area. It is noteworthy that publications related to ferroptosis in diabetes commenced in 2017, and the momentum of research appears unabated, suggesting a likely continuation of growth in the foreseeable future. As the pioneering study to undertake a bibliometric assessment in this domain, our work provides valuable insights into the trajectory of research and prospective avenues for exploration in the field of ferroptosis in diabetes. These findings offer robust support for both academic inquiry and clinical practice.

## Data availability statement

The original contributions presented in the study are included in the article/Supplementary material, further inquiries can be directed to the corresponding author.

## Author contributions

LX: Conceptualization, Data curation, Formal analysis, Software, Visualization, Writing – original draft, Writing – review & editing. FH: Conceptualization, Software, Visualization, Writing – review & editing, Methodology. ZL: Conceptualization, Investigation, Methodology, Writing – review & editing. XZ: Conceptualization, Methodology, Supervision, Writing – review & editing. YZ: Conceptualization, Funding acquisition, Resources, Supervision, Writing – review & editing.

## Glossary

DN: Diabetic nephropathy
DC: Diabetic cardiovascular
WOSCC: Web of Science Core Collection
NRF2: Nuclear factor erythroid 2-related factor 2
FPN1: Ferroportin1
ACSL4: Acyl-CoA synthetase long-chain family member 4
NF kappa b: Nuclear factor kappa b
NLRP3: NOD-like receptor family pyrin domain-containing 3
Sirt1: Silent information regulator 1
VADC1: Voltage-dependent anion-selective channel protein 1
DR: Diabetic retinopathy
I/RI: Iischemia/reperfusion injury
SLC7A11: Solute carrier family 7 member 11
GPX4: Glutathione peroxidase 4
NOX1: NADPH oxidase 1
T2DM: Type 2 diabetes mellitus
ROS: Reactive oxygen species
Drp1: Dynamin-related protein 1
AMPK: AMP-activated protein kinase
CD74: Cluster of differentiation 74
NAD: Nicotinamide adenine nucleotide
SIRT3: Sirtuin-3
SOD2: Superoxide dismutase 2
HO-1: Heme oxygenase 1
XC: Cystine/glutamate Antiporter system XC
Trx: Thioredoxin
