# Supplementary material for: The research trends of ferroptosis in diabetes: a bibliometric analysis
Source: Front Public Health. 2024 Mar 6;12:1365828. doi: 10.3389/fpubh.2024.1365828 (PMC10951384; doi:10.3389/fpubh.2024.1365828)
Supplement: Supplementary file 1 [file Table_1.DOC]

Supplementary Material

**Search terms and formulas**

**1** TS=(diabetes) OR (diabetic) OR (diabetic mellitus) OR (diabetes mellitus) OR (type 1 diabetes mellitus) OR (type 2 diabetes mellitus) OR (gestational diabetes mellitus) OR (pregnancy-induced diabetes) OR (aged diabetics ) OR (senile diabetes) OR (geriatric diabetes）OR (diabetes insipidus)

**2** TS= (high glucose) OR (hyperglycemic) OR (Glycemic) OR (glucose intolerance) OR (blood glucose) OR (blood sugar)

**3** TS=(diabetes neuropathy) OR (diabetic mononeuritis) OR (diabetic mononeuropathy) OR (diabetic neuritis) OR (diabetic neuropathies) OR (diabetic peripheral neuropathy) OR (diabetic polyneuritis) OR (diabetic polyneuropathy) OR (diabetic neuropathy) OR (painful diabetic neuropathy) OR (diabetic neuralgia) OR (diabetic autonomic neuropathy) OR (DPN)

**4** TS= (diabetic angiopathy) OR (diabetic vascular disease) OR (diabetic vascular complication) OR (diabetic microangiopathy) OR (diabetic cardiomyopathy)

**5** TS= (diabetic nephropathy) OR (diabetic kidney disease) OR (diabetes nephropathy) OR (diabetic nephrosis) OR (diabetic glomerulosclerosis)

**6** TS= (diabetic retinopathy) OR (diabetic retinopathies ) OR (diabetes retinopathy)

**7** TS= (diabetic foot) OR (diabetic feet) OR (foot ulcer, diabetic)

**8** TS=(diabetes complication) OR (diabetes-related complication) OR (diabetic complication)

**9 =** #1 OR #2OR #3 OR #4 OR #5 OR #6 OR #7 OR #8

**10** TS=(ferroptosis) OR (ferroptotic)

**#9 AND #10**
